# A Longitudinal Assessment of the Impact of Biological Maturity and Menarche on Adolescents’ Organized Sport and Physical Activity Participation

**DOI:** 10.1002/jad.12512

**Published:** 2025-05-08

**Authors:** Sharan Srinivasa Gopalan, Siobhan O'Dean, E. Jean Buckler, Sam Liu, Lauren A. Gardner, Katrina Champion

**Affiliations:** ^1^ School of Exercise Science, Physical and Health Education University of Victoria Victoria British Columbia Canada; ^2^ Matilda Centre for Research in Mental Health and Substance Use University of Sydney Sydney New South Wales Australia

**Keywords:** adolescents, biological maturity, menarche, organized sport, physical activity, puberty

## Abstract

**Introduction:**

Processes accompanying growth and maturation are known to impact physical activity (PA) participation among adolescents. This study evaluated the longitudinal impact of these processes on organized sport participation and moderate‐to‐vigorous PA (MVPA) among male and female adolescents.

**Methods:**

This study used secondary analysis of data from a longitudinal cohort of 6639 adolescents (Age = 12.92 ± 0.81 years; Males = 3302; Females = 3226) collected using confidential, online self‐report surveys through the Health4Life Study across 71 secondary schools in Australia from 2019 to 2022. Controlling for age, socioeconomic status, and state, mixed effects regression models assessed the impact of pubertal stage, relative pubertal timing, and period status (female adolescents only) on organized individual and team sports and MVPA participation.

**Results:**

While organized sport and MVPA participation reduced over time for all participants, male and female adolescents responded differently to puberty. Male adolescents showed marginally greater likelihood of participation in organized team sport during early puberty and individual sport during late puberty, but female adolescents significantly reduced individual sport participation during middle, late, and postpubertal stages. Relative pubertal timing did not impact male adolescents, but early maturing female adolescents were significantly less likely to participate in organized team and individual sports. Achieving menarche was linked to lower odds of individual sport participation only.

**Conclusions:**

Declining PA participation rates suggest that adolescents may need support for maintaining PA. Disparate effects of puberty in male and female adolescents advocate for separate approaches based on their specific characteristics. Future research should explore the impact of specific sport environment characteristics and menstrual cycle experiences on PA.

## 1 Introduction

Approximately 80% of the global adolescent population is insufficiently active (Van Sluijs et al. 2021). This is consistent with recent Australian data that indicates that only 20% of Australian children and youth meet the national guidelines for physical activity (PA), that is, 60 min of moderate‐to‐vigorous PA (MVPA) per day, with roughly 30% not involved in any weekly organized sport or physical activity (Hesketh et al. 2023). Physical inactivity has been identified as one of six critical lifestyle risk factors related to physical and mental health disorders, which tend to develop in adolescence and persist through adulthood (Uddin et al. 2020). PA, defined as any bodily movement that uses energy (World Health Organization 2022), has the potential to improve physical indicators of health and prevent conditions such as musculoskeletal issues and cardiovascular disease in children, adolescents, and adults (Rhodes et al. 2017). PA and organized sport participation are also associated with improved mental health outcomes and lower rates of psychological distress during adolescence (Ahn and Fedewa 2011; Ames et al. 2024; Panza et al. 2020; Smout et al. 2024). Furthermore, through the creation of strong peer relationships and support structures, PA and sport participation can also lead to improved social wellbeing and positive connections among adolescents (Pierannunzio et al. 2022).

Adolescence represents a time of considerable change for youth, with the adolescent growth spurt and puberty producing a multitude of physical, psychological, and social transformations (Manna 2014). In addition to the evolutionary predisposition towards reduced PA during adolescence, these changes can produce further obstacles and challenges related to PA and sport participation (Cumming et al. 2012). In a comprehensive review of the literature, Moore et al. (2020) found a modest inverse relationship between biological maturity and PA, with 50%‐60% of the included studies showing a reduced likelihood for PA and recreational sport participation among male and female adolescents who were more mature. Despite modest associations due to variations in data collection methods (i.e., subjective vs. objective) used for measuring PA and maturity (Moore et al. 2020; Sherar et al. 2010), these studies suggest the significance of pubertal stage (i.e., pre‐puberty, early/mid/late puberty, or postpuberty) and relative pubertal timing (i.e., early, average, or late maturing in relation to peers) to understanding PA behaviors among adolescents. Female adolescents also tend so show greater reductions in PA as compared to male adolescents during this stage, suggesting the presence of unique factors that impede their participation in activities (Telford et al. 2016). In addition to the challenges related to self‐esteem and body‐image among early maturing female adolescents, teasing and other negative interactions with peers can lead to further disengagement from PA (Cumming et al. 2012; Hazen et al. 2008; Slater and Tiggemann 2011).

Organized sport, both recreational and competitive, provides an effective means to engage youth in PA and teach sports skills while aiding in the development of important character and psychosocial traits (Beni et al. 2017; Hebert et al. 2015). However, the adolescent growth spurt can present challenges to sport participation with sudden changes in size and stature leading to higher sport dropout rates among adolescents (Crane and Temple 2015). Further, those lagging in their maturational development, and therefore less physically mature compared to their more developed peers, tend to be selected out of competitive youth sport and, in particular, team sports (Malina et al. 2015). While specific sports, for example, youth soccer, have been commonly included in assessments of puberty and sport participation factors, such as team selection and injury risk (Malina et al. 2021; Ribeiro et al. 2024), longitudinal evaluations of organized sports (individual and team), in general, could provide a more comprehensive view. Current evidence, albeit minimal, suggests that while organized sport participation decreases during adolescence, there is no significant association of this decline with pubertal stage or relative pubertal timing (Maia et al. 2010; Moore et al. 2020). A significant caveat among these findings from longitudinal analyses, however, is the lack of control for participant age (Moore et al. 2020). Further, the predominantly male population in these studies limits the generalizability of findings to broader populations. Given the lack of support and prevalent societal barriers related to gender norms that female adolescents face for PA and sport participation (Duffey et al. 2021), there is a need to address this gap and expand the analysis to this population.

Menarche, or the occurrence of the first period, can present an additional challenge. Menarche, and subsequent periods, can be accompanied by physical discomfort and social anxiety related to visible bleeding, leading to a lack of motivation or willingness to participate in social situations, including PA and organized sport (Srinivasa Gopalan et al. 2024). Complications arising due to irregular menstruation are also associated with a greater likelihood of physical inactivity among adolescents (Finne et al. 2011). Moreover, the societal taboo against menstruation, challenges with procuring menstrual hygiene products, and issues with tight and light‐colored athletic uniforms and apparel have the potential to exacerbate these problems (Srinivasa Gopalan et al. 2024). However, there is limited longitudinal research assessing the impact of period status across adolescence and its association with sport dropout tendencies among female adolescents (Guthold et al. 2020; Hopkins et al. 2022). Conducting such a longitudinal assessment could help to create a more holistic picture of PA and sport participation behaviors among adolescents.

To address these gaps, this study uses existing longitudinal data, over a 3‐year period, from a cohort of Australian adolescents to assess (1) the impact of pubertal status, relative pubertal timing, and period status on organized sport participation (team and individual), and (2) the impact of period status on moderate‐vigorous physical activity (MVPA), while controlling for participant age in all analyses. With differences in adolescents’ experiences, social dynamics, and dropout rates between individual and team sports, it is plausible that factors related to growth and maturation also impact sport participation in different ways in these environments (Back et al. 2022; Pluhar et al. 2019). Additionally, given the different trends in PA and sport participation among male and female adolescents, potentially indicating disparate effects of maturation and the environment, the analyses were conducted separately in these populations.

## 2 Methods

### 2.1 Study Design

We conducted a secondary analysis of longitudinal data from the “Health4Life” study, a cluster randomized controlled trial (RCT) of an eHealth lifestyle and behavior‐change intervention for adolescents. The trial was conducted in adolescents (age range 11–14 years) across 71 secondary schools in the three Australian states of New South Wales (NSW), Queensland (QLD), and Western Australia (WA) from July 2019 to December 2022. This trial is registered with the Australian New Zealand Clinical Trials Registry (ACTRN12619000431123). Ethics approval for this study was obtained from the University of Sydney (2018/882), the University of Queensland (2019000037), Curtin University (HRE2019–0083), and relevant ethics committees of the participating schools. Champion et al. (2023) and Teesson et al. (2020) provide complete details of the intervention and study protocol. Data were collected annually using confidential, online self‐report surveys administered to students with parent consent in a classroom setting under teacher supervision. Data from both trial groups, that is, control and intervention, were used in the present analysis, given that the intervention was not associated with significant effects on MVPA or light PA at any follow‐up time point (Champion et al. 2023; O'Dean et al. 2024). This study analyzed data collected at baseline (T1), 12 months (T3), 24 months (T4), and 36 months (T5). Data from post‐intervention, i.e., 6 weeks from baseline (T2), was not included as this period was considered too short to be of significance from a growth and maturation standpoint.

### 2.2 Measures

#### 2.2.1 Demographics

Participants’ age at each time point (i.e., baseline to 36 months) was calculated from their self‐reported date of birth and the date they completed each survey. Participants could indicate their sex at birth as “male” or “female” or choose to not disclose. As described in Champion et al. (2023), socioeconomic status (SES) was computed as low, medium, or high in relation to the study sample, and based on participants’ responses to the Family Affluence Scale (FAS; Torsheim et al. 2016).

#### 2.2.2 Organized Sport Participation

Participation in organized team sports (e.g., basketball, football, netball) or individual sports (e.g., little athletics, martial arts, dance) were evaluated through separate self‐report questions. Participants answered “yes” or “no” for their participation in organized individual or team sports for the duration of a school term or an entire sporting season.

#### 2.2.3 Moderate‐to‐Vigorous Physical Activity (MVPA)

MVPA participation was evaluated based on the Australian 24 h Movement Guidelines for Children and Young People, which recommends a minimum of 60 min of MVPA per day for children aged 5–17 years (Okely et al. 2022). Participants were provided with an explanation of MVPA as “activity that increases heart rate and gets you out of breath some of the time” and asked to recall the number of days over the past week where they believed they engaged in MVPA for more than 60 min during the course of the day. This single item MVPA recall measure has shown acceptable test‐retest reliability (Cohen's Kappa, κ = 0.503–0.599) among adolescents (Ng et al. 2019).

#### 2.2.4 Growth and Maturation Variables

Pubertal stage was computed based on the Pubertal Development Scale (PDS; Petersen et al. 1988). The PDS is a nonintrusive, reliable, and validated measure of pubertal development for adolescents (Koopman‐Verhoeff et al. 2020), consisting of six questions assessing changes in height, body hair, skin conditions, breast development, and menstruation. Pubertal stage was computed as a categorical variable, with levels pre‐pubertal (1), early pubertal (2), mid‐pubertal (3), late pubertal (4), and postpubertal (5). Period status was coded as “1” (achieved menarche or started to have their period) or “0” (not yet achieved menarche), based on female participants’ responses to the final question in the PDS questionnaire. Relative pubertal timing, that is, early, average, or late maturing, was computed as a relative status based on the position of an individual's score on the standardized distribution of total PDS scores.

### 2.3 Statistical Analysis

Frequency distributions were computed for all variables to capture the spread of participant responses. Dichotomous response variables, organized team sport and individual sport participation, were analyzed using a generalized linear mixed model (GLMM) fit to a binomial distribution. All analyses were performed in R (version 4.3.1) using the lme4 package. Given good‐to‐excellent reliability (Intraclass Correlation Coefficient, ICC = 0.694–0.765) of the single‐item MVPA recall measure as a continuous variable (Ng et al. 2019), this measure was treated as such and analyzed using a linear mixed model (LMM). Pubertal stage, relative pubertal timing, and period status, that is, the primary predictors, were included in the respective models as categorical independent variables. Participant age (continuous), study time point (continuous), state (categorical), and SES (categorical) were included in the model as additional fixed effects and control variables. A nested random intercept structure, with students nested within schools, was included in every model to account for the cluster randomized design of the Health4Life study. Model fit was compared using Akaike information criterion and Bayesian information criterion. All analyses were performed separately for male and female participants. Results of the GLMM analysis, if significant, are reported as odds ratios (OR), which indicates the change in the odds of the behavior related to the response variable for each unit change in the levels of the predictor variable. Results of the LMM analysis, if significant, are reported as the estimated mean change in the outcome in response to a unit change in the predictor. All results were supported with corresponding 95% confidence intervals (CI) and p‐values, with the significance level set at 0.05.

#### 2.3.1 Missing Data

Missing data analysis was conducted using binary logistic regression and T‐test for the binary‐outcome organized sport variables and MVPA recall variable, respectively, comparing differences in outcomes between those who completed only the baseline survey and those with one or more follow‐up survey responses (Champion et al. 2023; O'Dean et al. 2024; Smout et al. 2024).

## 3 Results

### 3.1 Participant Characteristics

A total of 6639 adolescents (Age = 12.92 ± 0.81 years) were present at baseline, of which 5525 (83.22%), 5012 (75.49%), and 4443 (66.92%) completed the 12‐, 24‐, and 36‐month follow‐up surveys, respectively. The study sample was fairly balanced between 3302 male (49.74%) and 3226 female participants (48.59%), with 94 participants (1.42%) choosing not to indicate their sex at birth. The majority of participants were from NSW (53.26%) and belonged to families of medium‐to‐high SES (76.89%), which was reflective of the population spread and demographics of Australia. Organized team sport showed a gradual decrease from 75.23% and 77.03% at baseline among male and female participants, respectively, to 41.52% and 41.07% at 36 months. Similarly, organized individual sport participation declined steadily from 57.87% and 70.8% at baseline among male and female participants, respectively, to 32.62% and 33.66% at 36 months. The number of days that male participants participated in 60 min of MVPA in a week only showed minimal reductions over 3 years, while that for female participants reduced from 4 days to about 3 days. Table 1 provides the full baseline characteristics of the study sample based on sex, SES, and state, while frequency distributions of the predictor and outcome variables are provided in Table 2.

**TABLE 1:** Participant characteristics.

Characteristic	*N* (%)
Participants	6639
Sex at birth	
Male	3302 (49.74)
Female	3226 (48.59)
Prefer not to say	94 (1.42)
Missing	17 (0.26)
State	
New South Wales	3536 (53.26)
Queensland	1788 (26.93)
Western Australia	1315 (19.81)
SES	
Low	909 (13.69)
Medium	2209 (33.27)
High	2896 (43.62)
Missing	625 (9.41)

**TABLE 2:** Frequency distribution of predictor and outcome variables.

Variable	Sex ‐ Male				Sex ‐ Female				Sex ‐ Prefer not to say			
	Baseline	12 months	24 months	36 months	Baseline	12 months	24 months	36 months	Baseline	12 months	24 months	36 months
Pubertal status n(%)												
1	173 (5.24)	58 (1.76)	26 (0.79)	31 (0.94)	70 (2.17)	11 (0.34)	2 (0.06)	5 (0.16)	—	—	—	—
2	521 (15.78)	217 (6.57)	61 (1.85)	26 (0.79)	120 (3.72)	22 (0.68)	5 (0.155)	3 (0.09)	—	—	—	—
3	1232 (37.31)	986 (29.86)	745 (22.56)	433 (13.11)	978 (30.32)	337 (10.45)	104 (3.22)	25 (0.78)	—	—	—	—
4	423 (12.81)	684 (20.72)	982 (29.74)	1098 (33.25)	1069 (33.14)	1396 (43.27)	1402 (43.46)	1068 (33.11)	—	—	—	—
5	9 (0.27)	16 (0.485)	35 (1.06)	99 (3)	84 (2.6)	159 (4.93)	356 (11.04)	634 (19.65)	—	—	—	—
Missing	944 (28.59)	1341 (40.61)	1453 (44)	1615 (48.91)	905 (28.05)	1301 (40.33)	1357 (42.06)	1491 (46.22)	—	—	—	—
Pubertal timing n(%)												
Average	966 (29.26)	955 (28.92)	784 (23.74)	808 (24.47)	962 (29.82)	906 (28.08)	712 (22.07)	771 (23.9)	17 (18.08)	28 (29.79)	20 (21.28)	19 (20.21)
Early	1127 (34.13)	694 (21.02)	687 (20.81)	578 (17.51)	1365 (42.31)	1104 (34.22)	1182 (36.64)	965 (29.91)	54 (57.45)	25 (26.6)	26 (27.66)	20 (21.28)
Late	1187 (35.95)	1028 (31.13)	964 (29.19)	795 (24.08)	880 (27.28)	681 (21.11)	535 (16.58)	423 (13.11)	21 (22.34)	20 (21.28)	13 (13.83)	10 (10.64)
Missing	22 (0.67)	625 (18.93)	867 (26.26)	1121 (33.95)	19 (0.59)	535 (16.58)	797 (24.71)	1067 (33.08)	2 (2.13)	21 (22.34)	35 (37.23)	45 (47.87)
Achieved Menarche n(%)												
Yes	—	—	—	—	1385 (42.93)	1902 (58.96)	2065 (64.01)	1938 (60.07)	—	—	—	—
No	—	—	—	—	1459 (45.23)	504 (15.62)	1641(4.99)	62 (1.92)	—	—	—	—
Missing	—	—	—	—	382 (11.84)	820 (25.42)	1000 (30.99)	1226 (38)	—	—	—	—
Organized individual sports n(%)												
Yes	1911 (57.87)	1432 (43.37)	1201 (36.37)	1077 (32.62)	2284 (70.8)	1758 (54.5)	1371 (42.5)	1086 (33.66)	55 (58.51)	40 (42.53)	30 (31.92)	19 (20.21)
No	1250 (37.86)	1147 (34.74)	1120 (33.92)	1017 (30.8)	847 (26.26)	891 (27.62)	960 (29.76)	1007 (31.22)	30 (31.92)	32 (34.04)	26 (27.66)	28 (29.79)
Missing	141 (4.27)	723 (21.9)	981 (29.71)	1208 (36.58)	95 (2.95)	577 (17.89)	895 (27.74)	1133 (35.12)	9 (9.57)	22 (23.4)	38 (40.43)	47 (50)
Organized team sports n(%)												
Yes	2484 (75.23)	1895 (57.39)	1590 (48.15)	1371 (41.52)	2485 (77.03)	1991 (61.72)	1613 (50)	1325 (41.07)	61 (64.89)	50 (53.19)	31 (32.98)	27 (28.72)
No	678 (20.53)	685 (20.75)	732 (22.17)	725 (21.96)	647 (20.06)	658 (20.4)	718 (22.26)	725 (21.96)	24 (25.53)	22 (23.4)	25 (26.6)	20 (21.28)
Missing	140 (4.24)	722 (21.87)	29.68)	1206 (36.52	94 (2.91)	577 (17.89)	895 (27.74)	1206 (36.52)	9 (9.57)	22 (23.4)	38 (40.43)	47 (50)
MVPA n(%)												
No. of days > 60 min in past 7 days	4.4 ± 2.11	4.39 ± 2.11	4.35 ± 2.19	4.24 ± 2.19	4.1 ± 1.93	4.03 ± 1.91	3.76 ± 2.02	3.36 ± 1.98	4.07 ± 2.21	4.38 ± 1.93	4.53 ± 2.25	3.9 ± 2.28

### 3.2 Outcomes From Mixed Model Analyses

The results of the mixed model analyses are presented in Tables 3 and 4 for male and female participants, respectively. The following sections break down the results for each of the main outcomes.

**TABLE 3:** Results of mixed model analyses assessing impact of maturity variables on organized sport among male adolescents.

Characteristic	Range/Levels	Organized team sports OR (95% CI)	Organized individual sports OR (95% CI)
Time	0–36 months	**0.75 (0.65, 0.87)**	**0.86 (0.78, 0.96)**
Pubertal stage (PDS category)	1	—	—
	2	**1.61 (1.01, 2.54)**	1.33 (0.93, 1.89)
	3	1.35 (0.90, 2.04)	1.37 (0.99, 1.88)
	4	1.36 (0.89, 2.09)	**1.43 (1.03, 2.00)**
	5	1.07 (0.55, 2.08)	1.21 (0.71, 2.05)
*Model fit*	—	*AIC* = *7692.2;*	*AIC* = *9657.8;*
		*BIC* = *7781.9*	*BIC* = *9747.5*
Relative pubertal timing	Average	—	—
	Early	0.94 (0.80, 1.12)	0.89 (0.73, 1.08)
	Late	1.17 (0.99, 1.38)	1.06 (0.86, 1.30)
*Model fit*	—	*AIC* = *9674.5;*	*AIC* = *12146.9;*
		*BIC* = *9753.0*	*BIC* = *12225.4*
Missing data	—	1.14 (0.85, 1.51)	1.05 (0.82, 1.35)

*Note:* First level/category of each predictor serves as the reference category. Significant results are indicated in bold. Akaike information criterion (AIC) and Bayesian information criterion (BIC) values provided for model fit.

**TABLE 4:** Results of mixed model analyses assessing impact of maturity variables on organized sport and MVPA among female adolescents.

Characteristic	Range/Levels	Organized team sports OR (95% CI)	Organized individual sports OR (95% CI)	MVPA ‐ Past 7 days estimate (95% CI)
Time	0–36 months	**0.66 (0.56, 0.77)**	**0.72 (0.63, 0.82)**	—
Pubertal stage (PDS Category)	1	—	—	—
	2	0.68 (0.26, 1.77)	0.55 (0.22, 1.38)	—
	3	1.01 (0.45, 2.27)	**0.45 (0.21, 0.97)**	—
	4	0.81 (0.36, 1.82)	**0.34 (0.16, 0.74)**	—
	5	0.67 (0.29, 1.56)	**0.39 (0.18, 0.88)**	—
*Model fit*	—	*AIC* = *7660.5;*	*AIC* = *8880.9;*	
		*BIC* = *7750.5*	*BIC* = *8970.9*	
Relative pubertal timing	Average	—	—	—
	Early	**0.82 (0.70, 0.96)**	**0.82 (0.71, 0.94)**	—
	Late	1.12 (0.92, 1.35)	1.18 (1.00, 1.39)	—
*Model fit*	—	*AIC* = *9693.5;*	*AIC* = *11247.6;*	—
		*BIC* = *9772.4*	*BIC* = *11326.4*	
Period status	0	—	—	—
	1	0.84 (0.69, 1.03)	**0.74 (0.63, 0.88)**	−0.08 (−0.18, 0.02)
*Model fit*		*AIC* = *9178.9;*	*AIC* = *10631.1;*	*AIC* = *36178.0;*
		*BIC* = *9249.9*	*BIC* = *10702.2*	*BIC* = *36256.2*
Missing data	—	**1.42 (1.07, 1.86)**	1.20 (0.92, 1.55)	t(3138) = 0.9318

*Note:* First level/category of each predictor serves as the reference category. Significant results are indicated in bold. Akaike information criterion (AIC) and Bayesian information criterion (BIC) values provided for model fit.

#### 3.2.1 Organized Team Sports Participation

The odds of participation in organized team sports declined over time for male (OR = 0.75, 95% CI = 0.65, 0.87, *p* < 0.001) and female participants (OR = 0.66, 95% CI = 0.56, 0.77, *p* < 0.001). Male adolescents in the early pubertal stage showed marginally greater odds of organized team sports participation (OR = 1.61, 95% CI = 1.01, 2.54, *p* = 0.043), while pubertal timing showed no significant association. For female adolescents, early maturing individuals had a reduced likelihood (OR = 0.82, 95% CI = 0.70, 0.96, *p* = 0.016) of organized team sports participation, with pubertal stage and period status showing no impact.

#### 3.2.2 Organized Individual Sports Participation

The odds of participating in organized individual sports declined over time for male (OR = 0.86, 95% CI = 0.78, 0.96, *p* = 0.009) and female participants (OR = 0.72, 95% CI = 0.63, 0.82, *p* < 0.001). Male participants in the late pubertal stage, however, showed greater odds of organized individual sports participation (OR = 1.43, 95% CI = 1.03, 2.00, *p* = 0.034), while pubertal timing showed no significant association for males. Female participants showed reduced participation in organized individual sports in the mid‐pubertal (OR = 0.45, 95% CI = 0.21, 0.97, *p* = 0.043), late‐pubertal (OR = 0.34, 95% CI = 0.16, 0.74, *p* = 0.006), and postpubertal (OR = 0.39, 95% CI = 0.18, 0.88, *p* = 0.022) stages. Early maturation was also significantly associated with reduced organized individual sports participation (OR = 0.82, 95% CI = 0.71, 0.94, *p* = 0.004). Period status, likewise, showed a significant association with organized individual sports, with those having undergone menarche less likely to participate in individual sports over time (OR = 0.74, 95% CI = 0.63, 0.88, *p* < 0.001).

#### 3.2.3 MVPA

Similar to both team and individual organized sports, MVPA participation declined over time (Estimate = ‐0.08, 95% CI = ‐0.18, 0.02, *p* < 0.001), but period status had no significant influence on the number of days per week that participants engaged in at least 60 min of MVPA.

#### 3.2.4 Missing Data

Compared to baseline‐only female participants, those who completed one or more follow‐up surveys had greater odds of organized team sport participation (OR = 1.42, 95% CI = 1.07, 1.86, *p* = 0.013). No additional significant differences were found for the predictors among male or female participants. Full results of the missing data analysis are included in Tables 3 and 4.

## 4 Discussion

This study examined the longitudinal associations between biological maturation (pubertal stage, relative pubertal timing, and period status) and participation in organized team sports, organized individual sports, and adherence to MVPA guidelines among adolescents over 3 years. Building on previous research, this study controlled for participant age, state of residence, SES, and time in conducting separate analyses for male and female adolescents to explore and uncover any sex‐based differences. In conducting longitudinal assessments for female adolescents and exploring the impact of menarche on PA and organized sport participation, these results contribute new findings to the growing literature. Overall, organized sport participation declined over time for both male and female adolescents, mirroring the global trends regarding general decline in PA in this population (Aubert et al. 2021). Pubertal stage, relative pubertal timing, and period status had a mixed impact on PA and organized sport behaviors, with trends mirroring past research with male and female adolescents, but modest strengths of association in these relationships suggest the intersection of various challenges, social conditions, and individual characteristics that could impact adolescents’ behaviors. The clear differences between male and female adolescents, however, suggest the need for acknowledging the varied impact of the maturation process in these populations.

Growth in body size and stature have the potential to produce challenges in coordination and physical skill performance for adolescents, but male adolescents can be expected to benefit from the increases in strength, power, and athletic ability as a result of these changes (Brown et al. 2017; Moore et al. 2020). It is unsurprising, therefore, that among male adolescents in this study, organized sport participation showed a predominantly positive trend in relation to pubertal stage, albeit with only results during early puberty for team sports and late puberty for individual sports reaching statistical significance. While improved participation in the latter stages of puberty could be attributed to improved athletic and technical capabilities (Brown et al. 2017), the lack of sustained improvement in organized team sport participation after the initial increase is surprising. One explanation is that male adolescents face more challenges with respect to dropout in team environments, especially with varying levels of physical maturity and development among their peers (Crane and Temple 2015). Typically, more mature adolescents would be able to dominate their lesser‐developed peers as a result of greater size and stature, often resulting in challenges for those lagging in their development. It is, therefore, surprising that pubertal timing did not show any significant association with male adolescents’ organized sport participation in this cohort. A potential reason could be the nature of the organized sport environment itself, with more competitive environments and coaches typically exerting greater pressure on adolescents, leading to disengagement from sport participation and greater dropout rates (Gardner et al. 2017). Additionally, understanding the type of sports that these adolescents engaged in could further inform the interpretation of these findings, with improvements in size and stature having specific implications based on the characteristics of the sport (Malina et al. 2015).

For female adolescents, changes accompanying growth and maturation have the potential to produce unique physical challenges as well as social awkwardness, negatively impacting their self‐concept and self‐efficacy for sport participation (Davison et al. 2007). In addition to insufficient education regarding the impact of their changing bodies on coordination and skill development, female adolescents undergoing these changes are also subject to teasing and derogatory comments from their peers and coaches (Slater and Tiggemann 2011). The lack of support and encouragement for female participants in sport and PA could further inhibit their desire and motivation to participate in PA and sport during this developmental phase, potentially impacting their future relationship with PA (Telford et al. 2016). These challenges were evident in relative pubertal timing showing a consistent inverse relationship with organized sport participation, with early maturing individuals less likely to participate in organized team sports and individual sports over time. The consistent negative impact of pubertal stage, especially the middle and latter stages, on organized individual sport participation among female adolescents in this study further points to the adversities that these experiences produce for young, developing female individuals. While the lack of association of pubertal stage with organized team sport participation is surprising, this could be a consequence of female adolescents receiving empathy, comfort, and support from their peers and older teammates undergoing similar experiences (Srinivasa Gopalan et al. 2024).

Period status, that is, achieving menarche, had an anticipated negative impact on sport participation, specifically in individual sports. In these environments, in particular, the male‐dominated nature of sport could produce greater challenges for female adolescents to discuss their symptoms and experiences related to the menstrual cycle, which are sensitive and private topics (Bergström et al. 2023). The lack of awareness and knowledge among coaches, typically male, and their reluctance to discuss the menstrual cycle with young female individuals leads to further silence on the topic in sport (Höök et al. 2021). An important caveat associated with these findings in this study, however, is the lack of information regarding participants’ individual challenges with menstruation. While irregular menstruation, heavy menstrual bleeding, and greater severity of menstrual symptoms negatively impact sport training and competition (Bruinvels et al. 2016; Srinivasa Gopalan et al. 2024), there is significant variability in the experiences related to the menstrual cycle and their impact on sport between and within female individuals (Schmalenberger et al. 2021). The lack of impact of menarche on MVPA in this sample, although surprising, could be a consequence of low initial rates of MVPA in this sample, with a large proportion of adolescents not meeting MVPA guidelines during the 3‐year period (Champion et al. 2023; Gardner et al. 2022; O'Dean et al. 2024).

In addition to the low levels of MVPA among adolescents, the declining trends in organized sport participation and the varied impact of pubertal development and maturation among male and female adolescents, as uncovered in this study, stress the need for persistent but nuanced approaches in supporting their sport and PA behaviors. Youth participating in competitive sport environments are routinely subject to high levels of pressure and expectations (Logan et al. 2019), often leading to challenges with mental health and sport dropout among adolescents (Crane and Temple 2015; Lang 2010). Therefore, it is imperative for organized sport environments to create inclusive and welcoming spaces that facilitate enjoyment and acknowledge and cater to the specific needs of adolescents undergoing puberty (Gano‐Overway and Guivernau 2018). The results from this study also highlight the need to challenge the patriarchal and male‐oriented culture of sport, with female adolescents needing specialized care and support in relation to the unique process that their bodies undergo and their relationship with sport and PA (Goorevich and LaVoi 2024). With the lack of resources and appropriate facilities to help female individuals manage menstruation, the inability to receive the appropriate support and empathy could lead to female adolescents developing negative associations with sport and PA participation (Srinivasa Gopalan et al. 2024).

Controlling for participant age and other confounding factors, such as state and SES, in a large longitudinal sample represent a particular strength of the analysis and findings from this study. However, specific characteristics of the data collection process and subsequent analysis warrant consideration in the interpretation of the study findings. First, while the survey differentiated between organized individual and team sports, the respective settings for these sport types is unknown, limiting the potential for generalizability across organized sport environments. For example, while some disciplines, such as track and field and swimming, would be classified as individual sports, training for the same could be conducted in group settings, thereby creating social dynamics akin to team sports (Evans et al. 2012). A more detailed exploration of the sport setting with additional details, such as the competitive versus recreational nature of the environment, the level of competition, and coach‐athlete dynamics, could shed more light on the relationship between puberty and organized sport participation among adolescents. Second, with the 12‐ and 24‐month assessments occurring during the COVID‐19 pandemic, it is important to acknowledge the influence of restrictions imposed by the pandemic on these findings, despite potential protection due to controlling for time and state in the analyses. Third, challenges with accuracy and validity of self‐report measures of pubertal development, like the PDS, as compared to clinician ratings and objective measures (Rasmussen et al. 2015), suggest the need for caution in the interpretation of these findings. Complementing the PDS responses with objective measures (e.g., skeletal age measurements) or estimation (e.g., age at peak height velocity) could help to produce more robust associations (Malina et al. 2015). Finally, the use of only subjective MVPA measures also serves as a limitation, with subjective measures typically not as accurate and relating to different correlates of PA, as compared to objective measures (Kavanaugh et al. 2015; Sirard and Pate 2001). Complementing this data with objective measurements (Gorzelitz et al. 2020), through wearable trackers, for example, could help to form clearer associations between biological maturity and PA and sport participation.

## 5 Conclusion

Declining rates of participation in organized sport and MVPA for male and female adolescents signal a need to focus efforts to help adolescents stay engaged in and enjoy PA and sport participation. However, as shown in this study, the support needs of male and female adolescents could differ, requiring a nuanced and custom approach due to the varied impacts of pubertal growth and development. While male adolescents showed marginal improvements during early and late puberty for team sport and individual sport participation, respectively, female adolescents’ individual sport participation reduced significantly as they underwent puberty, with team sport unchanged. Relative pubertal timing did not seem to impact male adolescents in sport, but early maturing female adolescents were consistently less likely to participate in organized team or individual sports. This study further highlighted the interaction between the menstrual cycle and sport participation, with the achievement of menarche negatively impacting organized individual sport participation. Future research should focus on identifying specific characteristics of the sport environment, such as social dynamics, pressures, and competition level, and individual experiences with the menstrual cycle for female adolescents, with respect to symptoms and symptom severity, to build on these findings. Complementing subjective measures with objective measures could further solidify findings.

## Ethics Statement

Ethics approval for this study was obtained from the University of Sydney (2018/882), the University of Queensland (2019000037), Curtin University (HRE2019–0083), and relevant ethics committees of the participating schools.

## Conflicts of Interest

The authors declare no conflicts of interest.

## Data Availability

The data that support the findings of this study are available from the corresponding author upon reasonable request.
